# Co-located Retail Clinics and Pharmacies: An Opportunity to Provide More Primary Care

**DOI:** 10.3390/pharmacy7030074

**Published:** 2019-06-26

**Authors:** Katherine Knapp, Keith Yoshizuka, Debra Sasaki-Hill, Rory Caygill-Walsh

**Affiliations:** 1 College of Pharmacy, Touro University California, 1310 Club Drive, Vallejo, CA 94592, USA; 2 Zuckerberg San Francisco General Hospital, 1001 Potrero Ave, San Francisco, CA 94702, USA

**Keywords:** community pharmacy, retail clinics, pharmacists, nurse practitioners, interprofessional training, primary care, healthcare access

## Abstract

This paper proposes that co-located retail clinics (RCs) and community pharmacies can increase opportunities to provide more accessible, affordable, and patient-friendly primary care services in the United States. RCs are small businesses of about 150–250 square feet with a clientele of about 10–30 patients each day and most frequently staffed by nurse practitioners (NPs). Community pharmacies in the U.S. at ~67,000 far outnumber RCs at ~2800, thereby opening substantial opportunity for growth. Community pharmacies and pharmacists have been working to increase on-site clinical services, but progress has been slowed by the relative isolation from other practitioners. An ideal merged facility based on an integrated platform is proposed. NPs and pharmacists could share functions that fulfill documented consumer preferences and still maintain separate practice domains. Potential benefits include a broader inventory of clinical services including laboratory tests, immunizations, patient education, and physical assessment, as well as better patient access, interprofessional training opportunities, and economies related to the use of resources, day-to-day operations, and performance metrics. Challenges include the availability of sufficient, appropriately trained staff; limitations imposed by scope of practice and other laws; forging of collaborative relationships between NPs and pharmacists; and evidence that the merged operations provide economic benefits beyond those of separate enterprises.

## 1. Purpose

We propose that the growing presence of co-located retail clinics (RCs) and pharmacies in the United States provides an opportunity to improve the delivery of accessible, affordable, and patient-friendly primary care services in the United States. In this paper, we present the case for the gains that could be made and the challenges that could reduce benefits and/or stand in the way of continued, successful implementation.

## 2. Methods

A three-person team with practice and research experience searched PubMed, Google Scholar, and Google for articles and papers about retail clinics, community pharmacy practice trends, training and clinical activities of nurse practitioners and pharmacists, primary care delivery trends, and approaches to merging business operations. Search terms included community pharmacy, retail clinics, pharmacist, nurse, and primary care. Previously collected resources were added to relevant articles. Based on the importance of legal issues, we only considered papers from the United States. The resource articles and follow-up discussions were used to identify benefits and challenges in establishing a successful, merged operation in co-located RCs and community pharmacies.

## 3. Background

### 3.1. Retail Clinics

The retail clinic (RC) is a relatively new site for providing primary healthcare services. These ambulatory sites render preventive and therapeutic services and are generally staffed by nurse practitioners (NPs) or physician assistants (PAs) [1,2,3]. In 2018, RC numbers in the U.S. were estimated at 2800, and the numbers continue to grow [4]. The potential value of RCs was explored in a landmark 2009 study that provided evidence that the cost of a typical RC visit ($110) was significantly less than similar visits to physician offices ($166), urgent care centers ($156), or emergency departments ($570), while the quality of care was equivalent or better [5]. The RCs are generally co-located with pharmacies. To date, CVS and Walgreens have sponsored the greatest numbers of RCs. Also sponsoring RCs, either directly or by contract, are mass merchandise corporations such as Target and Walmart and some grocery operations [2]. Here also, co-location with pharmacies is generally the rule. To give an idea of the potential for expansion, in 2015, <3% of the approximate 67,000 pharmacies in the U.S. had co-located RCs [6].

A 2015 report described RCs as small businesses of about 150–250 square feet with a clientele of about 10–30 patients each day [7]. It is hard to imagine RCs flourishing as independent, stand-alone entities; indeed, the business model pairing them with pharmacies has many potential advantages, a number of which we explore in this paper. With cost a critical issue for sustainability and expansion, it seems that co-located RCs and pharmacies should explore all reasonable economies by virtue of co-location.

### 3.2. Nurse Practitioners

NPs are generally preferred over PAs to lead RC operations. This is because scope of practice laws, which are generated at the state level, have allowed NPs in most states to have more autonomy from physician oversight. Given the small percentage of pharmacies co-located with RCs, a basic question for potential RC growth is the availability of sufficient numbers of appropriately prepared NPs.

Advanced Practice Registered Nurses (APRNs) provide diagnostic and therapeutic services for acute, episodic, or chronic illnesses [8,9]. APRNs include three cohorts, with NPs representing about 76% by number and nurse midwives and nurse anesthetists representing the rest. (Clinical nurse specialists are a fourth cohort of APRNs. This group is not currently represented in Bureau of Labor Statistics (BLS) data.) Bureau of Labor Statistics (BLS) data for 2017 report 166,280 employed NPs in the U.S. with mean annual wages of $107,480 [9]. There are several specialty areas for NPs based on the patient populations they are trained to care for. Some examples are pediatrics, acute care, psychiatry, and family care [10]. Family NPs (FNPs) are the only group licensed to treat both adults and children. This is an important consideration given that RCs treat both adult and pediatric patients, and it makes the FNP the best candidate for RC positions.

Requirements for licensure as an NP vary by state. Most states require RN status, completion of a Master’s degree or higher with emphasis on nursing skills from an accredited institution, and national certification from either the American Association of Nurse Practitioners or the American Nurses Credentialing Center [11]. Achieving NP status takes about two years beyond RN status. Nationally, there is a trend toward common licensure requirements for NPs. While every state would still require NP licensure to practice in that state, common licensure requirements could benefit RCs expanding across state lines by allowing NPs to gain licensure in another state more easily.

Health-related services that NPs can offer are determined by state-based scope of practice laws, a circumstance which has implications for RCs. Practice activities of NPs in California, for example, must meet the requirements for “standardized procedures” (SPs), meaning that only procedures that have been developed, reviewed, and approved by an interdisciplinary team can be legally performed [12]. Standardized procedures apply to diagnostic and treatment activities. One such procedure is the ability to prescribe medications under protocol. (In California, the term “furnish” is used in legal language regarding prescribing activities for both NPs and pharmacists.) For RCs, a particular advantage of co-location with pharmacies is this authority—an approved practice activity which is generally broader than allowed for pharmacists. The ability to obtain prescriptions which can potentially be filled under the same roof has been cited as a preference by patients [13]. There are, however, limitations on prescribing activities which are discussed below. A new direction related to services that can be offered is reflected in a bill that has been introduced in the California legislature that would allow NPs and physician assistants to practice independent of a supervising physician [14,15].

### 3.3. Pharmacists

By way of comparison with NPs, pharmacists are greater in number. According to the BLS [16], 309,330 pharmacists were employed in the United States in 2017. Their mean annual wage was $121,710. To become a pharmacist, one must earn a Doctor of Pharmacy (PharmD) through a program accredited by the Accreditation Council of Pharmacy Education (ACPE). The PharmD program requires four years, sometimes three years, depending on the curriculum of the institution. Once graduated, two sets of exams must be passed to obtain licensure: the North American National Pharmacist Licensure Examination (NAPLEX) and the Multistate Pharmacy Jurisprudence Examination (MJPE) or a state-specific law exam. Some pharmacists will continue training by completing a residency or fellowship. Some will pursue board certification in a specialty area by passing an examination. Currently, there are 41,000 [17] pharmacists worldwide that are Board of Pharmacy Specialties (BPS)-certified in 11 specialty areas such as pharmacotherapy, geriatrics, ambulatory care, and cardiology.

Pharmacists have been working professionally and politically to increase clinical involvement in patient care, especially in areas that relate to primary care. Through education and training, as well as expansion of scope of practice laws, progress has occurred. Indeed, pharmacists have been identified as part of the solution to reducing the shortage of primary care providers in this country [18,19]. Some states have passed laws allowing qualified pharmacists to expand their scope of practice and a pathway has been created to support another level of licensure: Advanced Practice Pharmacist (APP). California, North Carolina, Minnesota, New Mexico, Oregon, and Washington now support APP licensure. However, despite these advances, community pharmacies’ physical locations, somewhat isolated from other healthcare providers, have slowed this progress. The prospect of pharmacists partnering with NPs whose practice privileges overlap—but usually extend beyond those of pharmacists—opens the door to expanding services as a merged, joint operation.

### 3.4. Pharmacies

Health services offered in pharmacies have been expanding since the early 2000s when immunizations started being offered widely at pharmacies. More recently, but only in some states, pharmacies became able to prescribe naloxone for opioid overdose, hormonal contraceptives, and medications recommended for international travel. Pharmacies are providing smoking cessation programs, and there has recently been a proposal for pharmacists to prescribe medications to prevent the transmission of HIV [20,21,22]. Ohio recently passed SB 265 to provide reimbursement to pharmacists for clinical services by the state Medicaid program [23]. This trend toward expanding health services in community pharmacies has continued with both CVS and Walgreens recently launching initiatives. For example, CVS has opened three concept stores in the Houston area featuring “HealthHUBs” that offer assistance with durable medical equipment, yoga classes, blood tests, and nutritional advice in addition to their RCs (MinuteClinics) and pharmacies. CVS also announced the availability of telehealth services through its MinuteClinics [24]. Additionally, Walgreens renovated a store in Deerfield, Illinois to house a “Health Corner” offering such services as vision care, lab tests, and hearing services [25]. These initiatives by pharmacy industry giants may be an indication of the future direction of community pharmacies. Most recently, health insurer Aliera signed an agreement with CVS’s MinuteClinics to provide their members “with access to MinuteClinic services at no cost and with no applicable co-pays or deductibles” [26].

Besides these new ventures by pharmacy corporations, there is evidence that customers/patients are looking for an expanded service package when they go to a pharmacy. Table 1 combines results from three studies about consumer preferences for pharmacy-based health services. The first of these is a 2017 study of over 9000 people ≥18 years of age [27]. The second is a 2018 study of >1000 U.S. adults ≥40 years of age [13]. The third is a 2018 study that showed agreement among pharmacists, physicians, and patients that “minor ailments” could be well treated in rural community pharmacy settings by pharmacists, NPs, and/or PAs [28]. In these studies, participants identified preferred services they would like to see in community pharmacies. Table 1 suggests that, under the right circumstances, NPs and pharmacists could share several of these functions and still maintain separate practice domains. The shared services could allow for flexibility depending on the demand for services in either the RC or the pharmacy. This topic is explored further in a later section.

## 4. An Ideal Co-located Facility

### 4.1. Advantages of an Integrated Platform

Imagine for a moment that RCs and pharmacies could develop an integrated platform for delivering health services that are both preventive and therapeutic and that could also address customer preferences. What might it look like? The preferences in Table 1 indicate that customers and patients want their access to health care to be uncomplicated, quick, and convenient. In our ideal facility, for example, a patient could use a single access point—electronic, telephonic, or otherwise—to make an appointment for an immunization or blood pressure check, to get answers to questions about medication interactions, and/or to schedule a diabetic foot examination. Both NPs and pharmacists are trained to offer these services, although NPs perform diagnostic procedures more frequently. So, depending on the issue and overall workload, the patient’s appointment could be with either an NP or a pharmacist or both. In an ideal situation, staff could be cross-trained in areas that do not require specialized licenses or certificates to fill in for the other service area to prevent delaying or denying services to the patient. For situations requiring prescription medications, patients would have the option of filling their prescription and receiving counseling a few steps away, although there are caveats about getting prescriptions filled that are discussed below. In short, an integrated RC/pharmacy platform could mimic, on a smaller scale, a well-established and reputable health maintenance organization where a patient could receive several different healthcare services under one roof.

### 4.2. Clinical Laboratory Tests

Regarding customer preferences around clinical tests, the Clinical Laboratory Improvement Amendments 1988 (CLIA) describe quality standards for testing in clinical laboratories. For >1400 test systems that are non-technical and unlikely to provide erroneous results, CLIA requirements have been waived. These include, for example, blood glucose testing, cholesterol and hemoglobin A1c measurement, and testing for the presence of HIV-1 and HIV-2. Both pharmacies and RCs can offer diagnostic tests that are CLIA-waived after obtaining a CLIA Certificate of Waiver (COW). An integrated operation under the same roof and address could allow a single COW for the facility—a valuable efficiency saving time and money. In like fashion, inventories of vaccines could serve both the pharmacy and the RC.

### 4.3. Interprofessional Training Opportunities

Both pharmacist and NP training programs struggle with meeting mandates for interprofessional training as part of their programs. Such training is now mandated by accreditation agencies for most health professions [30,31]. An integrated, co-located RC and pharmacy could provide an ideal site for NP and pharmacy students to work with other health professionals—and one another—as part of providing care and being part of a care team. From a training perspective, these sites could help schools achieve accreditation requirements. From an economic perspective, students could augment the amount of service that can be offered to patients. From a student perspective, there is the opportunity for concrete experience working as a team in a primary care arena. One caveat would be that it is probably optimal that a site used for training be “mature” in the sense that integration is operational and the staff are experienced in working as a team.

### 4.4. Other Advantages

Some other advantages that could be realized with an integrated platform include providing more flexibility with hours of operation, providing more and richer patient education, creating in-house consultation on clinical issues, and expanding the service list that a single facility can offer.

## 5. Challenges

### 5.1. NP Supply

Despite the several clinical, financial, and convenience advantages cited above, there are barriers to consider and overcome. First, adequate numbers of appropriately trained NPs are needed to staff RCs. As noted earlier, only Family NPs (FNPs) are licensed to provide care for children and adults and would therefore be preferred to staff RCs. Approximately 67% of the NP cohort are FNPs [32], which, using BLS data, would be about 110,000 persons, an attractive pool for RC recruitment. Moreover, the BLS projects the number of NPs to grow by 31% between 2016 and 2026, a growth rate which is “much faster than average” [9]. However, given that NPs must provide evidence of registered nurse (RN) status to qualify for NP licensure, a shortage of RNs in the U.S., reported as recently as 2019, is relevant to the issue of sustained supply [33]. The same report lists reasons for the RN shortage to include a shortage of faculty that limits enrollment, an aging population of RNs, workplace stress, and difficulties finding appropriate training sites [34]. Based on the relationship between RN and NP supply, sustained RN growth will be an important factor in having sufficient FNPs to staff RCs.

### 5.2. Collaborative Practice Agreements

This paper discusses the topic of RCs located within a pharmacy on a national basis. There are still a handful of states where collaborative practice for pharmacists is severely restricted (such as the state of New York, where pharmacist collaborative practice agreements are restricted to teaching hospitals). Even in those states that allow collaborative practice agreements, some are so restrictive that a separate agreement must be established for each patient. The strategy of inserting the NP as the provider in RCs definitely has advantages, but state legal restrictions could limit the applicability of this strategy.

### 5.3. Antitrust and Stark Laws

The management of prescription medications is another issue that is potentially challenging. While it may seem logical that a prescription generated by a RC co-located with a pharmacy be filled at that pharmacy, the patient always has the choice of where to get that prescription filled. Limiting that choice may possibly violate antitrust laws [35] and the Stark laws [34] regarding illegal referrals. The prescriber may not direct the patient to use a particular pharmacy, nor can a lease agreement contain any requirements or incentives regarding the number of prescriptions generated. The Stark law specifically states in 42 USC §1395nn(a)(1)(A) “…the physician may not make a referral to the entity (where there is a financial arrangement) for the furnishing of designated health services for which payment otherwise may be made under this subchapter”, and the definition of health services specifically lists outpatient prescription drugs [36].

### 5.4. Pharmacist Reimbursement

Reimbursement for clinical services provided by pharmacists could be an impediment to the business success of co-located RCs and pharmacies. While pharmacists may bill Medicare for certain services as “incident-to” a visit [37], efforts to pass legislation for direct reimbursement to pharmacists under Medicare Part B have failed to make it out of committee in Congress [36,38]. There are some signs of progress; however, these are not without complications. For example, in California, a bill passed both houses and was signed by the governor in 2016 authorizing payment to pharmacists by MediCal (Medicaid) for clinical services allowed by SB 493 [39]. However, it has taken MediCal three years to issue billing codes to allow pharmacists to bill for such services. On the positive side, Washington State was successful in passing a bill in 2015 requiring commercial health plans to enroll pharmacists as providers and pay for services permitted within their scope of practice [40], and, as noted earlier, the Ohio legislature recently passed a bill to provide reimbursement to pharmacists for clinical services by the state Medicaid program [23]. Some commercial health plans have taken the initiative to recognize and reimburse pharmacists for clinical services, but this is more the exception than the rule.

One area where pharmacists have found success is by contracting with Accountable Care Organizations (ACOs) created by the Patient Protection and Affordable Care Act [41] to work within their scope of practice to improve the quality of care, improve accessibility, and reduce costs. The ACO is based upon population management as opposed to fee-for-service, so there is incentive to provide improved-quality care while reducing costs. Overall, these fragmented and state-based approaches to pharmacy/pharmacist reimbursement for clinical services have not made a major breakthrough and can still obstruct efforts to expand services in pharmacies whether or not they are co-located with RCs.

### 5.5. Business Relationship between the RC and Pharmacy

The last challenge we present is based on the financial and operational arrangements between the RC and pharmacy. Current models may not allow crossover in financial areas including payroll, reimbursements, and cost of supplies. This practice effectively places the pharmacy in competition with the RC. We would propose that the best chance for the success of co-located facilities to be greater than the individual successes of each entity is to establish an integrated platform that merges the use of resources, the inventory of clinical services, rules for day-to-day operations, and performance metrics while paying attention to customer preferences. This merging will be no easy task and will rely heavily on the development and use of skills and methods involving collaboration. To the best of our knowledge, there is currently no model that achieves a sustainable, merged organization, but a recent study outlines the characteristics of such a model and actions that can build the needed collaborative relationships [42]. With the continued deficit in primary care access and affordability in the United States and the potential to use existing facilities to a broader extent, it seems reasonable to explore, experimentally implement, and assess a merged operation. We hope that the information and ideas presented here can promote efforts toward a model that capitalizes on the advantages of co-locating RCs and community pharmacies.

## 6. Summary

Retail clinics (RC) co-located with pharmacies have the potential to increase the delivery of primary care in the United States. The advantages in merging the two operations include the ability to address customer preferences, to expand clinical services, to achieve efficiencies in managing supplies and materials, and to create more interprofessional training sites. Challenges to achieving a successful merged operation include the limitations imposed by scope of practice and other laws, difficulties with pharmacist reimbursement for clinical services, a possible shortage of family nurse practitioners, and the development of a practice model that achieves these goals while being financially successful. At this point, while the concept of co-located RCs and pharmacies is young, it is recommended to develop, implement, and assess a model or models based on merged, integrated operations.

## Figures and Tables

**Table 1:** Customer/Patient preferences from three studies.

Clinical Service	More Specific Details	Deliverable by Pharmacist or Pharmacy	Deliverable by NP or RC
**Access by appointment or walk-in**	Ability to make an appointment for service(s)	Some	Some
**Preventive health services**	Cholesterol, blood pressure, diabetes, osteoporosis screening, immunizations, life style evaluation, vitamins and supplements, lung function	Sometimes *	Yes, in California (CA) with standardized procedures (SPs)
**Diagnostic testing**	Diabetes mellitus, lipid/cholesterol measurements, testing for common infections including flu, strep, hepatitis, tuberculosis, HIV and chemistry tests (urine, saliva, and blood)	Yes	Yes, in CA with SPs
**(Quick) OTC treatments and/or recommendations**	Allergies, skin rashes, cough and cold, gastro-intestinal issues, feminine issues, sleeping aids, first aid, eye and ear problems, analgesics	Yes	Yes, in CA with SPs
**Patient education**	Advice on prescriptions, Medicare plans, medication interactions, side effects, over-the-counter medications, Medication Therapy Management, patient safety, behavioral counseling	Yes	Yes
**Prescription ordering, availability, information**	Managing prescriptions, procurement, inventory, recalls, counseling	Yes	No
**Medical records**	Keep records on medications, patient profiles, e.g., allergies	Sometimes ** Depends	Yes
**Physical examinations**	Blood pressure, heart rate, breathing rate, extremities	Yes	Yes
**Medication services**	New prescriptions, refill reminders, delivery, counseling	Yes	Yes, in CA with SPs
**Immunizations**	Vaccinations: flu, pneumonia, zoster, travel medications	Yes	Yes, in CA with SPs
**Drug prescribing**	Oral contraceptives, smoking cessation, adjustment of dose	Yes ***	Yes, in CA with SPs
**Advice and monitoring**	Weight loss, diabetes, cholesterol, blood pressure	Yes	Yes

* Some screenings may be performed by the pharmacist, while others require specialized equipment not available in the pharmacy. ** In most cases, a patient’s electronic health record is not readily available to the pharmacist, and a patient’s electronic health record is not readily available to the NP. However, in some pharmacies affiliated with a medical care system, this information can be available to both parties. *** Pharmacist prescribing is dependent upon the setting (a provider who contracts with a licensed health care service plan with regard to the care or services provided to the enrollees of that health care service plan [29]) or the level of license of the pharmacist (Advanced Practice Pharmacist (APP)).
